# Identification of small compounds regulating the secretion of extracellular vesicles via a TIM4-affinity ELISA

**DOI:** 10.1038/s41598-021-92860-2

**Published:** 2021-06-29

**Authors:** Yunfei Ma, Takeshi Yoshida, Kazutaka Matoba, Katsuhiko Kida, Rito Shintani, Yingshi Piao, Jingchun Jin, Taito Nishino, Rikinari Hanayama

**Affiliations:** 1 WPI Nano Life Science Institute (NanoLSI), Kanazawa University, Kanazawa, Japan; 2 Department of Immunology, Graduate School of Medical Sciences, Kanazawa University, Kanazawa, Japan; 3 Nissan Chemical Corporation, Nihonbashi, Japan; 4 Cancer Research Center, Yanbian University Medical College, Yanji, China; 5 Key Laboratory of Pathobiology of High Frequency Oncology in Ethnic Minority Areas, State Ethnic Affairs Commission , Yanbian University, Yanji, China; 6 Key Laboratory of Science and Technology Department, Yanbian University, Yanji, China; 7 Key Laboratory of the Changbai Mountain, Yanbian University, Yanji, China; 8 Department of Internal Medicine of Yanbian University Hospital, Yanji, China

**Keywords:** High-throughput screening, Multivesicular bodies, ESCRT

## Abstract

Extracellular vesicles (EVs) are secreted from most cells and play important roles in cell–cell communication by transporting proteins, lipids, and nucleic acids. As the involvement of EVs in diseases has become apparent, druggable regulators of EV secretion are required. However, the lack of a highly sensitive EV detection system has made the development of EV regulators difficult. We developed an ELISA system using a high-affinity phosphatidylserine-binder TIM4 to capture EVs and screened a 1567-compound library. Consequently, we identified one inhibitor and three activators of EV secretion in a variety of cells. The inhibitor, apoptosis activator 2, suppressed EV secretion via a different mechanism and had a broader cellular specificity than GW4869. Moreover, the three activators, namely cucurbitacin B, gossypol, and obatoclax, had broad cellular specificity, including HEK293T cells and human mesenchymal stem cells (hMSCs). In vitro bioactivity assays revealed that some regulators control EV secretion from glioblastoma and hMSCs, which induces angiogenesis and protects cardiomyocytes against apoptosis, respectively. In conclusion, we developed a high-throughput method to detect EVs with high sensitivity and versatility, and identified four compounds that can regulate the bioactivity of EVs.

## Introduction

Nano-sized extracellular vesicles (EVs), including exosomes, are released from most cells and play important roles in cell–cell communication by transporting proteins, lipids, and nucleic acids. EVs have been studied widely in recent decades owing to their involvement in various physiological phenomena and pathological processes, including adaptive immune responses, neurodegenerative diseases, and tumor progression^1^. In particular, tumor-derived EVs promote tumor metastasis by altering the tumor microenvironment^2,3^, induce angiogenesis by directly or indirectly activating the vascular endothelial cells^4–6^, and suppress antitumor immunity by activating immune checkpoints^7^. Suppressing the effects of EVs may be a potential therapeutic strategy for several EV-related diseases^8^. EVs have also attracted attention as therapeutic agents. For example, EVs derived from mesenchymal stem cells (MSCs) have been shown to have a therapeutic effect on neurodegenerative diseases^9^. Engineered-EVs artificially loaded with effective proteins, nucleic acids, or small compounds have also been developed^10^. Therapies targeting or utilizing EVs have potential in many diseases; however, their clinical use has been limited by a delay in the development of EV regulators that can modulate EVs without inducing severe side effects.

To date, several compounds have been reported to regulate EV secretion. Monensin, A23187, and ionomycin can facilitate EV release by increasing the concentration of intracellular calcium ions^11–13^. GW4869, an inhibitor of neutral sphingomyelinase 2 (nSMase2 or SMPD3), suppresses EV secretion by inhibiting the development of ceramide-dependent multivesicular endosome (MVE)^14^. Bafilomycin, and other autophagy inhibitors targeting V-ATPase, can markedly increase EV secretion^15,16^. However, cells generate and secrete EVs via multiple pathways. Development of MVEs is mediated by at least three pathways: ubiquitin-dependent endosomal sorting complex required for transport (ESCRT) pathway^17,18^, ubiquitin-independent ESCRT pathway^19^, and ESCRT-independent pathway^14^. MVE trafficking is mediated by multiple Rabs, including Rab11, Rab27, and Rab35^20^. EV secretion caused by fusion of MVEs and plasma membrane is mediated by SNARE and MUNC13-4^21,22^. Therefore, the existing regulators are not enough to regulate EV secretion in multiple cell types studied in preliminary or clinical studies. Some studies have screened EV regulators by using cells overexpressing fusion proteins of exosome marker and reporter proteins, such as CD63-EGFP or CD63-nanoluciferase, to increase the EV signal^16,23^, because marker proteins loaded on EVs are difficult to be detected in high-throughput screens. However, overexpression of fusion proteins may affect the physiological function, and the screened compounds may regulate CD63-positive EVs but not all EVs.

TIM4 binds to phosphatidylserine (PS) with high affinity (K_d_ ≈ 2 nM) and was identified as a receptor involved in the uptake of EVs^24^. As PS is enriched in EVs from various cells^25^, we previously developed an efficient method of isolating EVs using TIM4-affinity beads^26^. In this study, we employed a TIM4 EV-ELISA system, which enabled the detection of native EVs with high sensitivity using multiple antibodies against EV markers. Furthermore, a drug-repositioning library containing compounds investigated in phase I clinical trials was screened to identify druggable EV regulators. Consequently, we identified several compounds regulating EV secretion, and showed that some could regulate the biological activities of EVs.

## Results

### The TIM4-ELISA screen identified nine EV-regulators from a library of 1567 compounds

**Figure 1:**
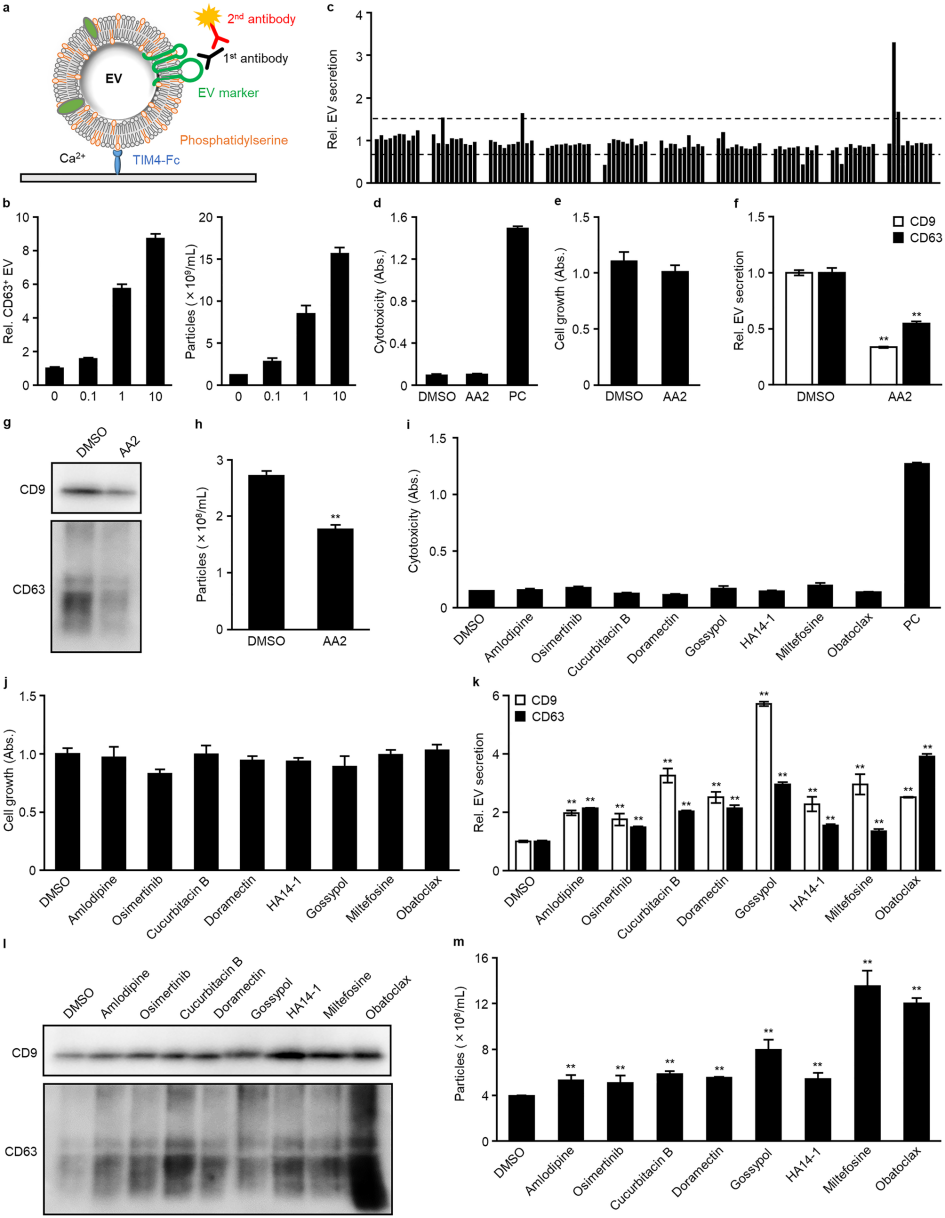
A TIM4-affinity ELISA screen in U87MG cells identified eight activators and one inhibitor of extracellular vesicles (EVs). (**a**) The principle of TIM4-ELISA. EVs are captured by immobilized TIM4-Fc proteins via calcium-dependent binding to phosphatidylserine on the surface of EVs. Then, the captured EVs are detected with a primary antibody against an EV surface marker, such as, anti-CD9, anti-CD63, or anti-CD81, and an HRP-conjugated secondary antibody. (**b**) K562 cells were stimulated for 24 h with 0.1, 1, or 10 μM monensin. EVs contained in the cultured supernatants were detected using TIM4-CD63 ELISA (left) and nanoparticle tracking analysis (NTA; right). (**c**) In the first round of screening, U87MG cells were treated with a 1,567-compound library at 0.1, 1, or 10 μM for 24 h. EVs contained in the cultured supernatants were detected using TIM4-CD63 ELISA. A typical result is shown here. The two dashed lines represent the threshold values for activators and inhibitors, at 0.67 and 1.5, respectively. (**d**–**m**) U87MG cells were treated with 10 μM AA2, 7 μM amlodipine, 2 μM osimertinib, 1 μM cucurbitacin B, 2 μM doramectin, 10 μM gossypol, 15 μM HA14-1, 20 μM miltefosine, or 1 μM obatoclax for 24 h. Cytotoxicity and cell growth were determined using lactate dehydrogenase (LDH) (**d**, **i**) and WST-8 (**e**, **j**) assays. Cells treated with lysis buffer were used as a positive control (PC) in the LDH assay. Secreted EVs were detected using TIM4-CD9 or TIM4-CD63 ELISA (**f**, **k**), Secreted EVs were recovered using ultracentrifugation and subjected to western blot with an anti-CD9 or anti-CD63 antibody (**g**, **l**), and NTA (**h**, **m**). Full-length blots can be found in the supplementary information. *p < 0.05, **p < 0.01, versus DMSO, Student’s *t*-test.

TIM4 binds to PS with high affinity, and has demonstrated the ability to capture PS-exposing EVs^26^. Here, we applied TIM4 to a sandwich ELISA system for a high-throughput chemical screen (Fig. 1a). The TIM4-ELISA showed good quantitative capability (Fig. 1b, CV = 8.25 and 3.46% at 0 and 10 µM, respectively, S/N = 8.70, R^2^ = 0.9832) in an experiment of monensin-dependent EV secretion from K562 cells^11^ and a higher sensitivity compared to conventional EV-ELISA (Supplementary Fig. S1). We prepared a drug-repositioning library containing 1,567 compounds, and screened EV regulators using the TIM4-ELISA by treating U87MG cells with three doses of each drug (0.1, 1, and 10 µM). A compound increasing the secretion of EVs by more than 1.5-fold or less than 0.67-fold was classified as an activator or inhibitor, respectively. After the first round of screening using the TIM4-CD63 ELISA, 38 and 22 compounds remained candidate EV activators and inhibitors, respectively (Fig. 1c). In the second round of screening, we examined the effects of the compounds on EV secretion using TIM4-CD9 and TIM4-CD63 ELISAs and evaluated the cytotoxicity of the compounds. Consequently, 18 and six compounds were able to induce or inhibit EV secretion without obvious cytotoxicity. In the third round of screening, in addition to the previous assessments, we also evaluated the concentration of EV particles by nanoparticle tracking analysis (NTA). Consequently, we identified an inhibitor, apoptosis activator 2 (AA2) (Table 1), which induced a 66.4 and 45.3% decrease in the secretion of CD9- and CD63-positive EVs, respectively, and a 35.1% decrease in the number of secreted EVs without affecting cell growth (Fig. 1d–f). Similar results were seen in the western blot of EVs isolated by TIM4-beads (Supplementary Fig. S2a). As the TIM4-capture methods are limited to PS-exposing EVs, we demonstrated the decrease in EV secretion by western blot of EVs isolated using ultracentrifugation or cultured supernatant without EV isolation. There was no clear difference between the EV isolation methods (Fig. 1g, Supplementary Fig. S2b). Moreover, the western blot results showed a similar tendency to those obtained by TIM4-ELISAs. NTA also revealed the reduction of EV particle number (Fig. 1h), and there was no apparent change in EV size compared to the DMSO group (Supplementary Fig. S3a). Overall, the data indicated that AA2 has ability to suppress secretion of EVs. We also identified eight EV activators: amlodipine, osimertinib, cucurbitacin B, doramectin, gossypol, HA14-1, miltefosine, and obatoclax (Table 1). The cytotoxicity assay and TIM4-ELISA demonstrated that these compounds induced a 1.3- to 5.7-fold increase in the secretion of CD9- or CD63-positive EVs without affecting the growth of U87MG cells (Fig. 1i–k). The results of the western blot analysis of EVs isolated by ultracentrifugation revealed that the compounds increased secretion of CD9- or CD63-positive EVs (Fig. 1l). Consistent results were observed in the western blot analysis of EVs isolated by an affinity isolation method using TIM4-beads and supernatants obtained by centrifugation at 10,000 × *g* (10 K sup) (Supplementary Fig. S2a,b). NTA revealed that the eight activators were able to increase the number of EV particles without affecting the size of the EVs (Fig. 1m, Supplementary Fig. S3b). In all experiments, the eight compounds enhanced EV release in all experiments, but the degree of increase differed depending on the method used. In particular, gossypol treatment resulted in a high intensity of CD9 and CD63 in the TIM4-ELISA study but only slight increases in the western blot analyses and NTA. HA14-1 treatment resulted in a significant increase of CD9 in the western blot but only a slight increase in the TIM4-ELISA. These inconsistencies may be caused by the differences in EV isolation methods. Miltefosine, which strongly induced EV secretion in the NTA, was associated with only slight increases in the EV-ELISA and western blot analyses, suggesting that miltefosine may induce the secretion of EVs sorted less proteins.

**Table 1:** Activity of EV regulators in U87MG cells.

Compounds	CAS No	CD9^+^-EVs in ELISA (%)	CD63^+^-EVs in ELISA (%)	EV particles in NTA (%)
DMSO	67-68-5	100.0	100.0	100.0
AA2	79183-19-0	33.6	54.7	64.9
Amlodipine	88150-42-9	197.5	213.8	133.9
Osimertinib	1421373-65-0	174.9	147.9	128.9
Cucurbitacin B	6199-67-3	325.6	202.7	148.1
Doramectin	117704-25-3	251.3	212.9	140.0
Gossypol	303-45-7	571.9	295.1	201.8
HA14-1	65673-63-4	227.6	154.2	137.0
Miltefosine	58066-85-6	295.5	134.1	342.7
Obatoclax	803712-67-6	251.3	390.8	304.2

We observed the effects of EV regulators on the cells using immunofluorescent staining of CD63. It was revealed that AA2 decreased intracellular CD63 expression. Moreover, the decrease in transcription of CD63 was lower than the decrease in intracellular CD63 expression (Supplementary Fig. S4a–c), implying that AA2 may suppress MVE development. Five activators, namely amlodipine, osimertinib, cucurbitacin B, miltefosine, and obatoclax, increased intracellular CD63 expression, whereas three activators, namely doramectin, gossypol, and HA14-1, did not (Supplementary Fig. S4a, b). In addition, none of the compounds increased the transcription of CD9 or CD63 (Supplementary Fig. S4c). These results imply that the former group may promote MVE development, whereas the latter may activate MVE transport or MVE-plasma membrane fusion.

### AA2 inhibited EV secretion independent of nSMase2 and caspase 3

**Figure 2:**
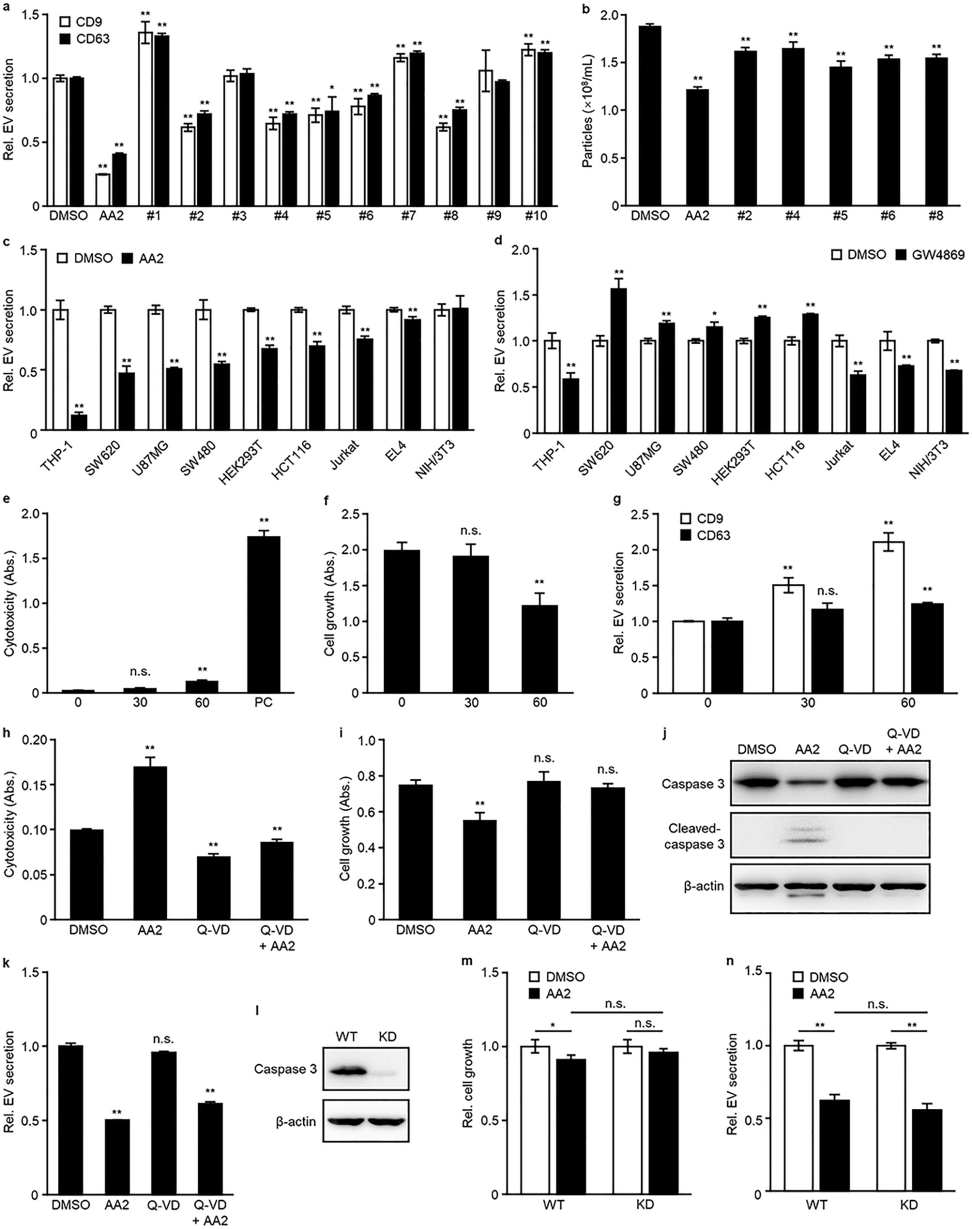
AA2 inhibited EV secretion independent of nSMase2 and caspase 3. (**a**, **b**) U87MG cells were treated for 24 h with #1, #7, or #10 at 1 μM; AA2, #2–#6, #8, or #9 at 10 μM, and then secreted EVs were determined using TIM4-CD9 or TIM4-CD63 ELISA (**a**) or NTA (**b**). (**c**, **d**) Cell lines were treated with AA2 as shown in Table 3 (**c**) or 3 μM GW4869 (**d**) for 24 h and then secreted EVs were determined using TIM4-ELISA. EVs secreted from THP-1, Jurkat, and EL4 cells were detected using TIM4-CD81 ELISA. EVs secreted from SW620, U87MG, SW480, HEK293T, HCT116, and NIH/3T3 cells were detected using TIM4-CD63 ELISA. (**e**–**g**) U87MG cells were treated with 30 or 60 μM MT-21 for 24 h. Cytotoxicity and cell growth were determined using LDH (**e**) and WST-8 (**f**) assays. Secreted EVs were determined using TIM4-CD9 or TIM4-CD63 ELISA (**g**). (**h**–**k**) Jurkat cells were pre-treated with 0 or 50 μM Q-VD for 3 h, and then with 0 or 5 μM AA2 for 24 h. Cytotoxicity and cell growth were determined using LDH (**h**) and WST-8 (**i**) assays. (**j**) The cells were lysed and immunoblotted with anti-caspase 3, anti-cleaved-caspase 3, or anti-β-actin antibody. Full-length blots can be found in the supplementary information. (**k**) Secreted EVs were determined using TIM4-CD81 ELISA. (**l**) HEK293T WT or *CASP3* KD cells were lysed and immunoblotted with anti-caspase 3 or anti-β-actin antibody. Full-length blots can be found in the supplementary information. (**m**, **n**) HEK293T WT or *CASP3* KD cells were treated with 3 μM AA2 for 24 h. Cell growth was determined using a WST-8 assay (**m**) and secreted EVs were determined in TIM4-CD81 ELISA (**n**). *p < 0.05, **p < 0.01, n.s.; not significant, versus DMSO or AA2-treated WT, Student’s *t*-test.

To investigate the structure–activity relationship of AA2, we evaluated the effects of 10 AA2 analogs (Table 2). Five analogs, (#2, #4, #5, #6, and #8) moderately affected EV secretion in TIM4-CD9, TIM4-CD63 ELISA, and NTA (Fig. 2a, b). AA2 and these effective compounds possessed two chlorines at the 3,4 position of the benzyl group or carried no halogen in the benzyl group. Notably, even the simplest analog (#8) demonstrated moderate activity. Modifications around the indole ring resulted in slight changes in the activity of the analogs. While compounds 1, #3, #7, #9, and #10, which presented no activity, possessed one halogen in the benzyl group or two chlorines at the 2,6 position of the benzyl group. These data indicated that the 3,4-dichlorobenzyl group in AA2 is essential for the inhibitory effect on EV secretion. Next, we evaluated the effect of AA2 on different human and mouse cell lines. Prior to the experiment, an optimal EV marker for each cell line was determined using TIM4-ELISA, and the most easily detectable marker was used in the subsequent experiments. Although the expression levels of EV markers cannot be simply compared because the different antibodies differ in affinities, the easily detectable marker was selected for each cell line (Supplementary Fig. S5). Consequently, AA2 inhibited the secretion of EVs in eight of nine cell lines, namely THP-1, SW620, U87MG, SW480, HEK293T, HCT116, Jurkat, and EL4 (Fig. 2c). A similar evaluation of GW4869, an inhibitor of EV secretion targeting nSMase2^14^, revealed that EV secretion was inhibited in four of nine cell lines, namely NIH/3T3, Jurkat, EL4, and THP-1 (Fig. 2d). As the cellular specificity of AA2 and GW4869 differ, AA2 may inhibit a pathway other than the nSMase2-dependent pathway.

**Table 2:**
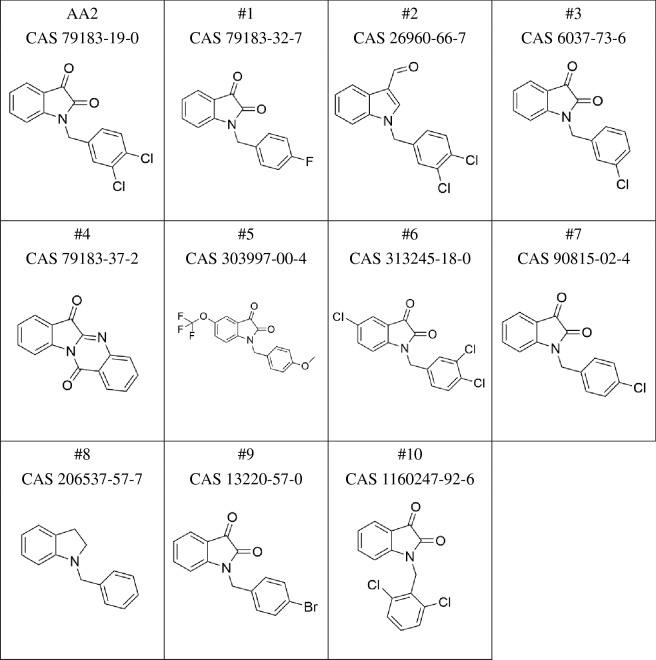
Structure of AA2 and its analogs.

**Table 3:** Concentrations of AA2, cucurbitacin B, gossypol, or obatoclax in different cells.

Cells	AA2 (μM)	Cucurbitacin B (μM)	Gossypol (μM)	Obatolax (μM)
THP-1	10	0.1	1	0.1
SW620	10	3	3	0.03
U87MG	10	1	10	1
SW480	10	0.3	3	0.1
HEK293T	3	1	0.3	0.03
HCT116	10	3	3	0.03
Jurkat	1	0.01	1	0.03
EL4	0.3	0.03	1	0.01
NIH/3T3	1	0.1	3	0.03
hMSC	n.t	1	10	1
K562	n.t	0.03	0.3	0.1

*n.t.* Not tested.

AA2 is a strong activator of caspase 3, which plays a key role in the execution phase of apoptosis^27^. However, in our study, AA2 inhibited EV secretion at concentrations lower than those that induced cell death (Fig. 1d–h). Thus, AA2 may suppress EV secretion independent of caspase 3-activation. To test this hypothesis, we evaluated the effect of MT-21, an activator of caspase 3, on EV secretion and showed that MT-21 failed to inhibit EV secretion without affecting cell growth (Fig. 2e–g). Furthermore, we used an irreversible pan-caspase inhibitor Q-VD-OPH or *CASP3* knock-down (KD) cells to exclude the involvement of caspase 3. Q-VD-OPH inhibited the cytotoxicity of high-concentrations of AA2 and restored the growth of Jurkat cells (Fig. 2h,i). Western blotting analysis for caspase 3-cleavage also showed that Q-VD-OPH inhibited caspase 3 activation induced by AA2 (Fig. 2j). Conversely, treatment with Q-VD-OPH did not affect the inhibitory effect of AA2 on EV secretion (Fig. 2k). Consistent results were obtained in treatment with another caspase 3 inhibitor, Z-VAD(OMe)-FMK, although it was less effective than Q-VD-OPH in recovering cell growth (Supplementary Fig. S6a–c). Similarly, AA2 inhibited EV secretion to a similar level in both HEK293T wild-type and *CASP3*-KD cells (Fig. 2l–n). These data indicated that the inhibition of EV secretion by AA2 is independent of caspase 3-activation.

### Cucurbitacin B, gossypol, and obatoclax induce EV secretion in a variety of cell lines

**Figure 3:**
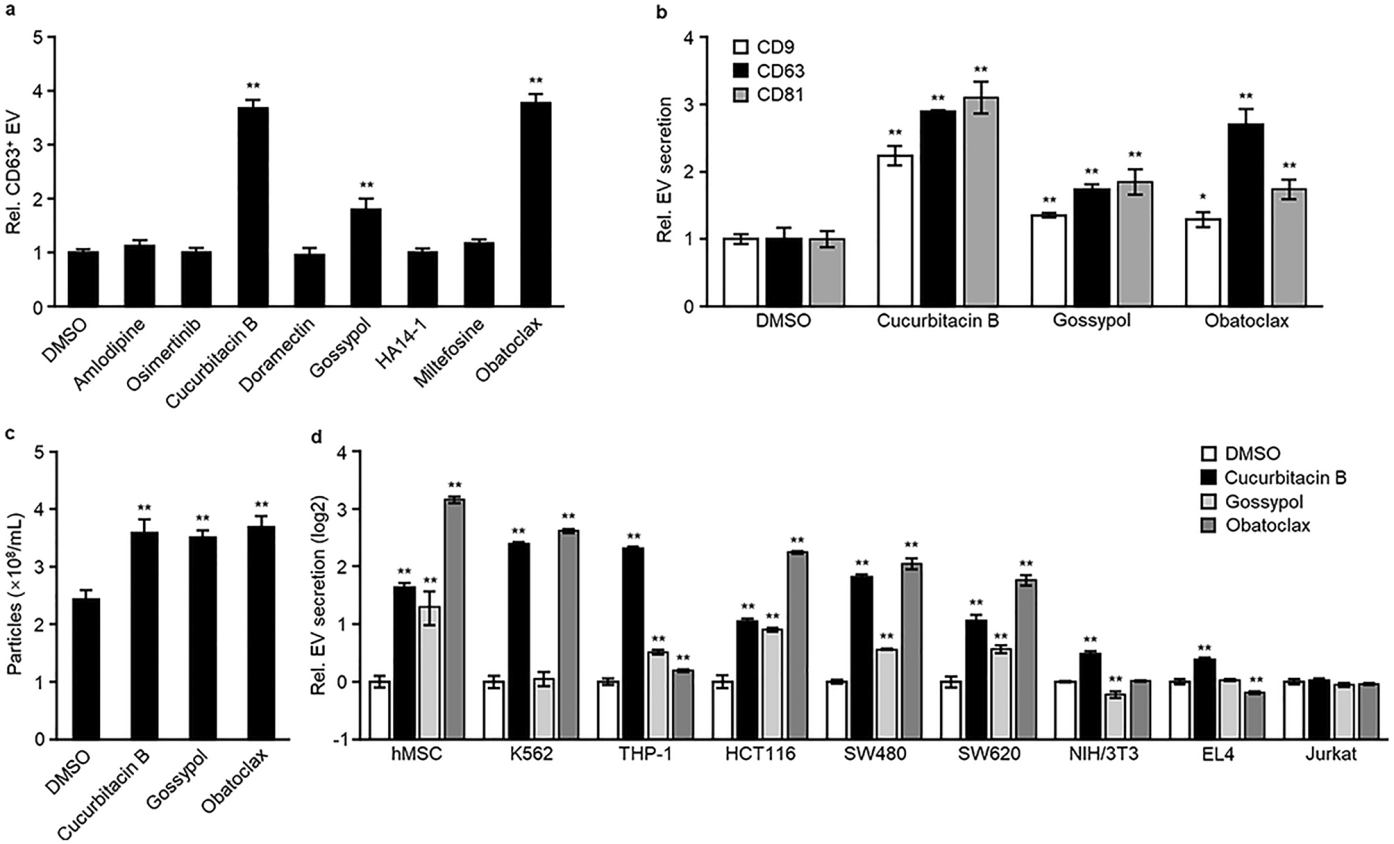
Cucurbitacin B, gossypol, and obatoclax induced EV secretion in a variety of cells. (**a**–**c**) HEK293T cells were treated with 1 μM amlodipine, 0.3 μM osimertinib, 1 μM cucurbitacin B, 1 μM doramectin, 0.3 μM gossypol, 10 μM HA14-1, 10 μM miltefosine, or 0.03 μM obatoclax for 24 h and then secreted EVs were determined using a TIM4-CD63 ELISA (**a**), TIM4-CD9, TIM4-CD63, TIM4-CD81 ELISA (**b**) or NTA (**c**). (**d**) Cell lines were treated with cucurbitacin B, gossypol, or obatoclax at the concentrations shown on Table 3 for 24 h, and then secreted EVs were determined using a TIM4-ELISA. EVs secreted from hMSC, K562, HCT116, SW480, SW620, and NIH/3T3 cells were detected using TIM4-CD63 ELISA. EVs secreted from THP-1, EL4, and Jurkat cells were detected using TIM4-CD81 ELISA. *p < 0.05, **p < 0.01, versus DMSO, Student’s *t*-test.

HEK293T and HEK293 cells are commonly used to prepare engineered EVs^28–30^. We evaluated the effect of eight EV activators on CD63-positive EV secretion in these cells. After determining the non-toxic concentration of each activator, cells were treated at the maximum non-toxic concentration. Cucurbitacin B, gossypol, and obatoclax induced CD63-positive EV secretion in both HEK293T and HEK293 cells (Fig. 3a, Supplementary Fig. S7a). These three activators also promoted the secretion of CD9- and CD63-positive EVs and increased the number of EV particles secreted from HEK293T and HEK293 cells (Fig. 3b,c and Supplementary Fig. S7a,b). Next, we tested these three compounds in a variety of cells. We found that cucurbitacin B induced EV secretion in all cells except for Jurkat cells, showing broad cellular specificity (Fig. 3d). Gossypol and obatoclax presented a slightly narrower cellular specificity. (Fig. 3d). No compounds induced EV secretion in Jurkat cells. These data indicated that the effect of activators on EV secretion is cell-type dependent.

### EV regulators have potential to regulate the bioactivity of EVs

**Figure 4﻿:**
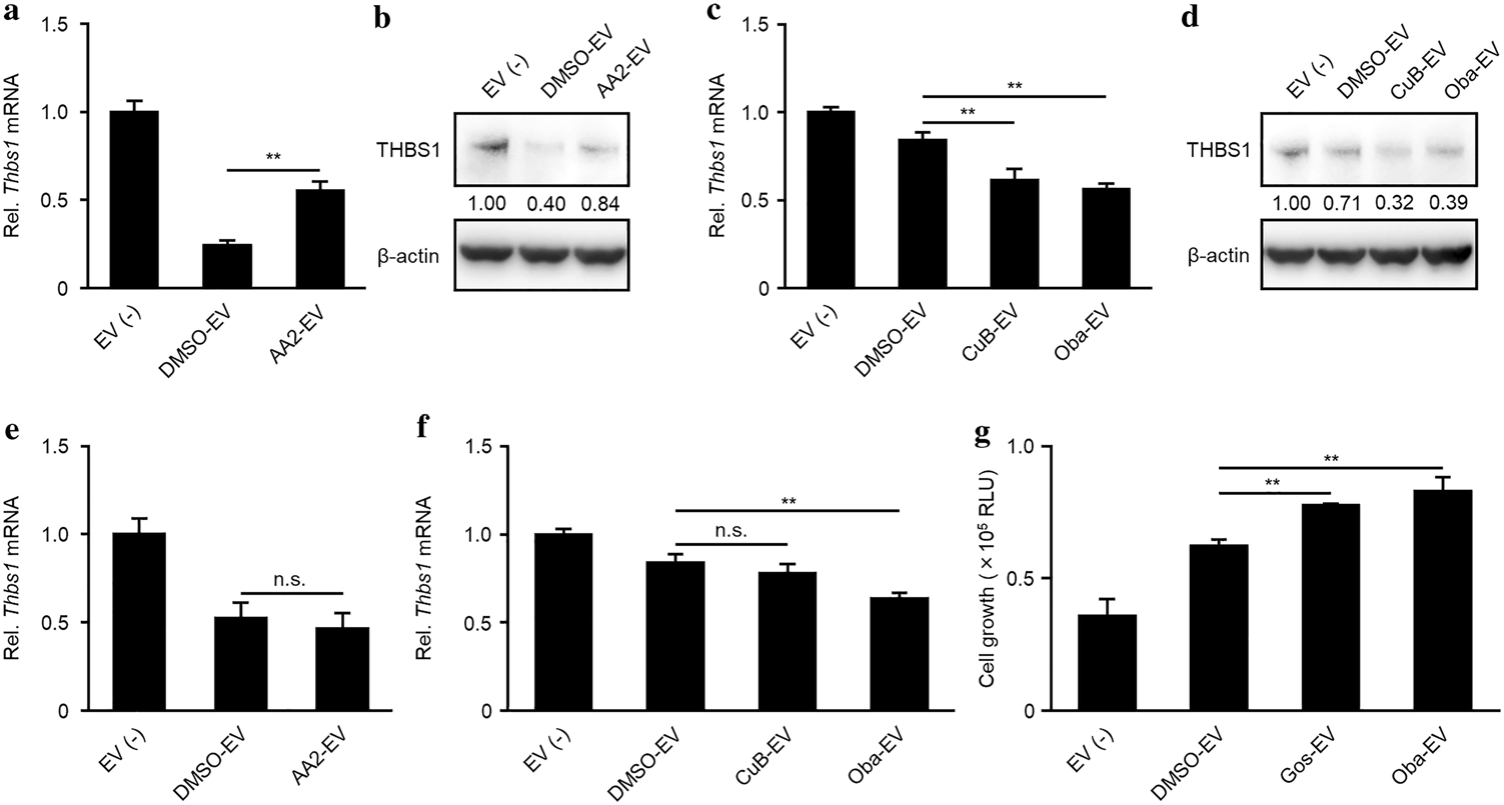
AA2, cucurbitacin B, gossypol, and obatoclax can regulate EV bioactivity in vitro. (**a**–**f**) DMSO-, AA2-, CuB-, or Oba-EVs were isolated from the cultured supernatant of U87MG cells following treatment for 2 days with DMSO, AA2, cucurbitacin B, or obatoclax. MG6 cells were stimulated with EVs isolated from the same number of U87MG cells (**a**, **b**; 1 × 10^7^ cells, **c**, **d**; 3 × 10^6^ cells) or with the same number of EV particles (**e**; 1 × 10^10^ particles, **f**; 3 × 10^9^ particles) for 12 h. Total RNAs or cellular proteins were extracted from MG6 and the expression of *Thbs1* was detected using RTqPCR (**a**, **c**, **e**, **f**) or western blot (**b**, **d**). **p < 0.01, n.s.; not significant, versus DMSO-EV, Student's *t*-test. A THBS1 band intensity in (**b**, **d**) was normalized to β-actin. Full-length blots can be found in the supplementary information. (**g**) DMSO-, Gos-, or Oba-EVs were isolated from the cultured supernatant of hMSCs following treatment for 2 days with DMSO, gossypol, or obatoclax. H9C2 cells pre-treated with 1 mM CoCl_2_ were stimulated with the EVs isolated from 4 × 10^5^ hMSCs for 4 days. Cell growth was measured via a luminescent cell viability assay. **p < 0.01, versus DMSO-EV, Student’s *t*-test.

Previous studies have demonstrated the contribution of tumor-derived EVs to angiogenesis in tumor progression^6,31–33^. In particular, EVs secreted from glioblastoma U87MG cells are transferred into microglia MG6 cells, where they downregulate the expression of thrombospondin 1 (*Thbs1*), a negative regulator of angiogenesis^6,34^. To clarify whether the EV regulators have the potential to regulate EV bioactivity, we treated U87MG cells with the EV regulators and evaluated the effect of EVs secreted from the cells on the expression of *Thbs1* in MG6 cells. EVs isolated from AA2-treated U87MG cells suppressed the downregulation of *Thbs1* expression in MG6 cells (Fig. 4a,b), and those from cucurbitacin B- or obatoclax-treated U87MG cells enhanced *Thbs1* downregulation (Fig. 4c,d). The increase or decrease in the effect of EVs in MG6 cells could be attributed to the regulation of the number of EVs secreted from U87MG cells or to changes in the quality of EVs. The regulation of the number of EVs by EV-regulators are shown in Fig. 1, but changes in the quality of EVs by EV-regulators has not yet been verified. Treatment of the identical number of EVs isolated from each EV-regulator-treated U87MG cells revealed that EVs from AA2- or cucurbitacin B-treated U87MG cells had a similar bioactivity to DMSO, and those from obatoclax-treated U87MG cells had a slightly higher bioactivity than DMSO (Fig. 4e,f). These data indicated that AA2, cucurbitacin B, and obatoclax can regulate the number of secreted EVs maintaining their quality. Additionally, we evaluated the ability of these EV regulators to protect cardiomyocytes against hypoxia-induced apoptosis by hMSC-derived EVs^35^. EVs from hMSCs treated with DMSO did not recover the growth of cardiomyocyte H9C2 cells, while those treated with gossypol or obatoclax significantly recovered the growth of H9C2 cells when the EVs from the same number of hMSCs were treated (Fig. 4g). These results suggested that the EV regulators may have therapeutic potential for EV-related diseases.

## Discussion

In this study, we screened compounds that could regulate EV secretion using a TIM4-affinity EV-ELISA system. Compared with the traditional EV-ELISA using anti-CD63 antibodies and so on, the TIM4-ELISA enables EVs to be captured with low-bias, and with tenfold higher sensitivity^26^. These characteristics are useful for high-throughput screenings on a small-scale, such as those performed in a 384-well plate, or detection following a short stimulation of no more than 24 h. As PS-exposing EVs are secreted from various cells^25^, the TIM4-ELISA will enable highly sensitive detection of EVs secreted from a variety of cells. It should be noted that the results obtained by TIM4-ELISA, western blot, and NTA were not completely consistent with each other (Fig. 1k–m, Supplementary Fig. S2). This may be because the TIM4-based EV capture methods, including the TIM4-ELISA and the affinity isolation method using TIM4-beads, capture only limited EV populations; (i.e., PS-exposing EVs). In particular, if a compound affects the amount of PS on EVs, the TIM4-based EV capture methods would not be appropriate for EV quantification. A second reason for the inconsistency is the difference in density between EVs isolated by the ultracentrifugation method and those isolated by the TIM4-based methods. It has been reported that EVs isolated by the ultracentrifugation method also include EVs with higher density than those isolated by the TIM4-based method^26^. As a third reason, the TIM4-ELISA results in inferior purity compared with the affinity isolation method using TIM4-beads. The TIM4-ELISA captures all molecules bound to TIM4-Fc, whereas the affinity isolation method using TIM4-beads captures TIM4-Fc-binding molecules and then isolates only those molecules eluted with EDTA. Because each EV analysis has advantages and disadvantages, it is important to evaluate the amount of EVs using multiple methods to obtain more precise information.

The inhibitor, AA2, inhibited EV secretion from a variety of cells in vitro, including tumor and non-tumor cells. However, considering the effect of AA2 on apoptosis, its use clinically for patients with EV-related diseases may be limited. Notably, the inhibitory effect of AA2 on EV secretion was observed with concentrations lower than those that induce apoptosis in most cells, and EV secretion was inhibited via a pathway independent of caspase 3. Therefore, it may be possible to develop an inhibitor that regulates EV secretion with no or limited effects on apoptosis. We were unable to identify such a compound in this study; however, we elucidated the structure–activity relationship of AA2 analogs. This information will be helpful for developing suitable AA2 derivatives.

Based on the NCBI Gene database (https://www.ncbi.nlm.nih.gov/gene/), *NSMASE2* mRNA is expressed in limited human and mouse tissues. We hypothesized that the narrow cellular specificity of GW4869 may be because of the limited expression of *NSMASE2*. Conversely, AA2 has wider cellular specificity, suggesting that AA2 may target a molecule expressed in various cell types. Overall, the different cellular specificities of AA2 and GW4869 might be helpful for inhibiting EV secretion in a variety of cells.

Datta et al. identified EV activators, including fenoterol, norepinephrine, N-methyldopamine, mephenesin, and forskolin, after screening a chemical library in prostate cancer cell lines^23^; however, these compounds did not have the desired effect in hMSCs^36^. Limited cellular specificity has also been observed for some EV regulators evaluated in this study; therefore, it is important to select the most effective activator for each cell type. Furthermore, it has been assumed that there are many pathways by which cargos are sorted onto EVs, and only a few have been uncovered^37^. In this study, EV regulators did not affect the ability of U87MG-EVs or hMSC-EVs to promote angiogenesis or inhibit hypoxia-induced apoptosis. However, they might affect the biological activity of EVs produced in other cells. In production of EVs loaded with functional molecules, it is important to confirm that treatment with EV-regulators does not alter a biological activity of EVs.

Notably, cucurbitacin B is a Bcl-2, Mcl-1, and Bcl-XL inhibitor^38^, and gossypol and obatoclax are pan-Bcl-2 family inhibitors^39^. These three compounds induce apoptosis by inhibiting members of the anti-apoptotic Bcl-2 family but are not known to regulate EV secretion. Members of the anti-apoptotic Bcl-2 family repress apoptosis by inhibiting Ca^2+^ release from the endoplasmic reticulum (ER), in so-called calcium induced apoptosis^40^. As the involvement of Ca^2+^ signaling in EV secretion has been reported in several cell lines^11,22^, it is possible that the release of Ca^2+^ from ER by the activators may induce EV secretion. Treatment with these activators at non-toxic concentrations may induce Ca^2+^ release at levels not sufficient to induce apoptosis, but sufficient to induce EV secretion. In addition, autophagy induced by these activators may enhance EV secretion, since members of the anti-apoptotic Bcl-2 family are autophagy suppressors^41^. Autophagosomes generally develop into autolysosome by fusing with lysosomes; however, by inhibiting this pathway, amphisomes are developed by fusing the accumulated autophagosomes with MVEs, which are then exported from the cells. The activators enhance the generation of autophagosomes and amphisomes, which may indirectly increase EV secretion.

Here, we identified several EV regulators through high-throughput screening of a 1,567-compound library. Capture of EVs by a high-affinity lipid binder and detection of EVs using multiple EV surface markers enabled the efficient identification of EV regulators. The EV regulators rarely change the quality of EVs but regulate the number of EVs secreted, presenting the desired characteristics for drug development. We tested the effects of EV regulators only in vitro; thus, there are still several issues to be clarified before they are used for the treatment of EV-related diseases, including the delivery system of EV regulators to target cells, effects on EV secretion from normal cells, and side effects. We hope that these regulators will contribute to the development of EV drugs or the therapy for EV-related diseases by resolving the issues.

## Methods

### Cells

U87MG (ATCC, Manassas, VA), HEK293 (Riken BRC, Ibaraki, Japan), HEK293T (Riken BRC), NIH/3T3 (ATCC), LM8 (Riken BRC), HCT116 (Riken BRC), SW480 (ATCC), SW620 (ATCC), and EL4 (Riken BRC) cell lines were maintained in Advanced DMEM (Thermo Fisher Scientific, Waltham, MA) supplemented with 2% heat-inactivated fetal bovine serum (FBS; Thermo Fisher Scientific), penicillin–streptomycin, and L-glutamine. K562 (Riken BRC), Jurkat (Riken BRC), and THP-1 (Riken BRC) cells were maintained in Advanced RPMI-1640 (Thermo Fisher Scientific) with 2% heat-inactivated FBS, penicillin–streptomycin, and L-glutamine. Human mesenchymal stem cells from adipose tissue (PromoCell, Heidelberg, Germany) were maintained in Cellartis MSC Xeno-Free Culture Medium (Takara Bio, Shiga, Japan) with Cellartis MSC Xeno-Free Supplement and used in experiments by maximum 10 passages. MG6 (Riken BRC) cells were maintained in high-glucose DMEM (Thermo Fisher Scientific) supplemented with 10% FBS, penicillin–streptomycin, 10 μg/mL insulin (FUJIFILM Wako Pure Chemical, Osaka, Japan), and 0.1 mM 2-Mercaptoethanol (Merck, Darmstadt, Germany). H9C2 (ATCC) cells were maintained in high-glucose DMEM supplemented with 10% FBS and penicillin–streptomycin. All cells were cultured at 37 °C with 5% CO_2_. To detect EVs, FBS was replaced with EV-free FBS, which was prepared by mixing 10 mL of heat-inactivated FBS with 2 mL of 50% PEG-10,000 (Merck) by rotating at 4 °C for 4 h. The supernatant was then collected after centrifugation at 2,000 × *g* for 20 min. HEK293T *CASP3* KD cells were established via the CRISPR/Cas9 system. In brief, a pX330Puro plasmid targeting a sequence in the human *CASP3* gene (GGAATGACATCTCGGTCTGG, PAM sequence is underlined) was transfected into HEK293T cells. After 3 days of selection with puromycin, CASP3 expression was determined via western blot analysis. All cell lines were tested for mycoplasma contamination by PCR targeting the 16S ribosome.

### Chemical treatment

A compound library was purchased from MedChemExpress (Monmouth Junction, NJ), and compounds more recently registered than CAS#124,508–13-0 and compounds with cytotoxicity were excluded from the library. U87MG cells were seeded in Advanced DMEM-2% EV-free FBS containing 0.03% SphereMax (Nissan chemical, Tokyo, Japan) into 384-well plate or plates of other sizes, and treated with a chemical library, ionomycin (Merck), monensin (Merck), or DMSO (Merck). Other cells were seeded in Advanced medium-2% EV-free FBS into 96- or 24-well plates, and treated with a chemical library, ionomycin, monensin, or DMSO. After incubating the cells for 24 h, the plate was centrifuged at 1,200 × *g* for 60 min to separate the conditioned medium (1.2 K sup) from the cells. A WST-8 assay (Nacalai Tesque, Kyoto, Japan) was used to evaluate cellular proliferation, according to the manufacturer’s protocols. To evaluate the amount of EVs or cytotoxicity, the 1.2 K sup was subjected in TIM4-affinity ELISA or LDH assay. The LDH assay (Dojindo, Kumamoto, Japan) was performed according to the manufacturer’s protocol. In experiments performed on a larger than 384-well scale, the conditioned medium was separated by serial centrifugation at 300 × *g* for 5 min, 2,000 × *g* for 20 min, 10,000 × *g* for 30 min. The supernatant resulting from centrifugation at 10,000 × *g*, 10 K sup, was used in TIM4-affinity ELISA, NTA, and for the isolation of EVs.

### TIM4-affinity ELISA

An ELISA plate (AGC Techno Glass, Shizuoka, Japan) was coated with 1 μg/mL recombinant mouse TIM4-Fc protein (FUJIFILM Wako Pure Chemical)/50 mM carbonate-bicarbonate buffer (pH 9.6) and incubated at 4 °C overnight. The wells were blocked with 1% bovine serum albumin (BSA)/2 mM CaCl_2_/0.05% tween20/TBS buffer (TBS-TCa) for 1 h. Then, 1.2 K sup or 10 K sup supplemented with 2 mM CaCl_2_ was applied to the wells, and the cells were incubated for 1 h at room temperature. The captured EVs were labeled with each primary antibody/TBS-TCa for 2 h and then each HRP-conjugated secondary antibody for 1 h at room temperature. Finally, EVs were detected with TMB reagent (Nacalai Tesque) by measuring the absorbance at 450 nm. Information regarding antibodies used in this study is listed in Table 4.

**Table 4:** Antibodies used in TIM4-ELISA or western blot.

Antibody, clone	Manufacturer, catalog #	Working concentration
**TIM4-ELISA**		
Human CD9, HI9a	BioLegend, 312102	× 1000
Human CD63, H5C6	BioLegend, 353039	× 1000
Human CD81, 5A6	BioLegend, 349502	× 1000
Mouse CD9, MZ3	BioLegend, 124802	× 1000
Mouse CD63, NVG-2	BioLegend, 143902	× 1000
Mouse/rat CD81, Eat-2	BioLegend, 104901	× 1000
Anti-mouse IgG HRP	BioLegend, 405306	× 5000
Anti-rat IgG HRP	BioLegend, 405405	× 5000
Anti-hamster IgG HRP	Jackson ImmunoResearch, 127035160	× 5000
**Western blot**		
Caspase-3	Cell Signaling Technology, 9662	× 1000
Cleaved Caspase-3 (Asp175)	Cell Signaling Technology, 9661	× 1000
β-actin, AC-15	Merck, A5441	× 5000
THBS-1 antibody, D7E5F	Cell Signaling Technology, 37879	× 1000
Anti-mouse IgG HRP	BioLegend, 405306	× 5000
Anti-rabbit IgG HRP	BioLegend, 406401	× 5000

### Western blotting

For detecting cellular proteins, cells were lysed in RIPA buffer [50 mM Tris–HCl (pH 8.0), 150 mM NaCl, 2 mM EDTA, 0.5% sodium deoxycholate, 1% TritonX-100, 0.1% sodium dodecyl sulfate] with protease inhibitor cocktail on ice for 20 min, and then centrifuged at 14,000 × *g*, 4 °C for 20 min to harvest the cell lysates. The cell lysates were separated by SDS-PAGE and transferred onto PVDF membranes. The membranes were blocked in 5% skim milk in 0.05% tween20/TBS buffer for 1 h, and then incubated with each primary antibody at 4 °C overnight, followed by each HRP-conjugated secondary antibody. Immunoblot signals were captured using the Image Quant Las 4000mini (GE Healthcare, Chicago, IL) and SuperSignal West Pico Chemiluminescent Substrate (Thermo Fisher Scientific). For detecting EV proteins, the 10 K sup was centrifuged at 100,000 × g for 2 h using an S50ST rotor (Eppendorf Himac Technologies, Ibaraki, Japan), and then washed in 7 mL PBS (−) by another centrifugation at 100,000 × g for 2 h. The EV pellet was lysed with 2 × sample buffer [100 mM Tris–HCl, pH 6.8, 20% (v/v) glycerol, 4% (w/v) SDS] and separated using SDS-PAGE. The subsequent steps were carried out in the same way as for the detection of cellular proteins. Information regarding antibodies used in this study is listed in Table 4.

### NTA

The 10 K sup was diluted with PBS (−) to an appropriate concentration, 1 × 10^8^–1 × 10^9^ EVs/mL, and the concentration of EVs was determined using NanoSight LM10 (Malvern Panalytical, Malvern, United Kingdom). In brief, approximately 600 μL of supernatant was loaded onto the sample stage and the movement of EVs was recorded at camera level = 15 and the same temperature for 30 s. Three different fields were recorded by advancing the sample. Data was analyzed using the NTA3.1 software at detection threshold = 3. The EV concentration was calculated by multiplying the dilution factor.

### Model of glioblastoma-derived EV-mediated angiogenesis

EVs were isolated from the 10 K sup of U87MG cells following treatment for 2 days with DMSO or EV regulators, using the MagCapture Exosome Isolation Kit PS (FUJIFILM Wako Pure Chemical) according to the manufacturer’s instructions. After isolating EVs, the buffer was replaced with PBS (−) in dialysis. The effect of U87MG-EVs was evaluated in a model of EV-mediated angiogenesis, as previously reported^6^. Briefly, MG6 cells were seeded at 1 × 10^5^ cells in a 24-well plate and cultured for 12 h. The cells were treated for 12 h with the indicated amounts of U87MG-EVs. After washing the cells with PBS (−), total RNA was extracted using RNeasy Plus Mini Kits (QIAGEN, Hilden, Germany). Single-strand cDNA was synthesized using ReverTra Ace qPCR master mix (TOYOBO, Osaka, Japan). *Thbs1* or *Gapdh* gene was amplified and detected using the LightCycler 96 (Roche, Basel, Switzerland) with Universal SYBR Select master mix (Thermo Fisher Scientific) and paired primers, *Thbs1*-Fw; 5′-CACCTCTCCGGGTTACTGAG-3′ and *Thbs1*-Rv; 5′-GCAACAGGAACAGGACACCTA-3′, or *Gapdh*-Fw; 5′- GTGTTTCCTCGTCCCGTAGA-3″ and *Gapdh*-Rv; 5′-AATCTCCACTTTGCCACTGC-3′.

### Hypoxia-induced cardiomyocyte apoptosis assay

To isolate EVs, 4 × 10^5^ hMSCs were cultured for 4 days and then treated for 2 days with DMSO or EV regulators. The EVs were isolated from 10 K sup of the hMSC-conditioned medium using the Capturem Exosome Isolation Kit (Takara Bio), according to the manufacturer’s instructions. After isolating EVs, the buffer was replaced with PBS (−) using the Amicon Ultra PLGC Ultracel column, 10 membrane, 3 kDa column (Merck). To induce hypoxia-induced apoptosis, 5 × 10^4^ H9C2 cells were cultured for 1 day in DMEM-10%FBS, followed by 1 day in DMEM-10%FBS containing 1 mM CoCl_2_ (FUJIFILM Wako Pure Chemical), and 4 days in DMEM-10%FBS containing the EVs from hMSCs treated with DMSO or EV regulators. The cells were used to perform a in CellTiter-Glo Luminescent Cell Viability Assay (Promega, Madison, WI) according to the manufacture’s protocol.

## Supplementary Information

Supplementary Information.

## Data Availability

The authors declare that all data supporting the findings of this study are available within the paper and the associated supplementary information files or from the corresponding author upon reasonable request.
