# Supplementary material for: Identification of small compounds regulating the secretion of extracellular vesicles via a TIM4-affinity ELISA
Source: Sci Rep. 2021 Jun 29;11:13471. doi: 10.1038/s41598-021-92860-2 (PMC8242084; doi:10.1038/s41598-021-92860-2)

Supplementary information

Identification of small compounds regulating the secretion of extracellular vesicles via a TIM4-affinity ELISA

Yunfei Ma[1,2], Takeshi Yoshida[1,2]*, Kazutaka Matoba[3], Katsuhiko Kida[3], Rito Shintani[1,2], Yingshi Piao[4,5,6,7], Jingchun Jin[4,5,6,7,8], Taito Nishino[3], Rikinari Hanayama[1,2]*
[1]WPI Nano Life Science Institute (NanoLSI), Kanazawa University, Kanazawa, Japan.
[2]Department of Immunology, Graduate School of Medical Sciences, Kanazawa University, Kanazawa, Japan.
[3]Nissan Chemical Corporation, Nihonbashi, Japan.
[4]Cancer Research Center, Yanbian University Medical College, Yanji, China.
[5]Key Laboratory of Pathobiology of High Frequency Oncology in Ethnic Minority Areas, State Ethnic Affairs Commission, Yanbian University, Yanji, China.
[6]Key Laboratory of Science and Technology Department, Yanbian University, Yanji, China.
[7]Key Laboratory of the Changbai Mountain, Yanbian University, Yanji, China.
[8]Department of Internal Medicine of Yanbian University Hospital, Yanji, China.

*Corresponding authors: Takeshi Yoshida and Rikinari Hanayama

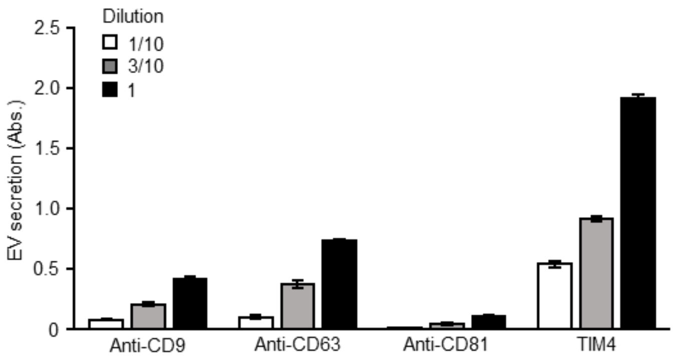

Supplementary Figure S1. The TIM4-ELISA increases the binding capacity of EVs. Anti-human CD9, anti-human CD63, or anti-human CD81 antibody or TIM4 protein was coated at 1 μg/mL on an ELISA plate. 10K sup of U87MG cells diluted by the indicated ratios were added to the plate. Bound EVs were detected with biotin-anti-human CD63 antibody (BioLegend, 353017, ×1000), followed by HRP-conjugated streptavidin (BioLegend, 405210, ×3000).

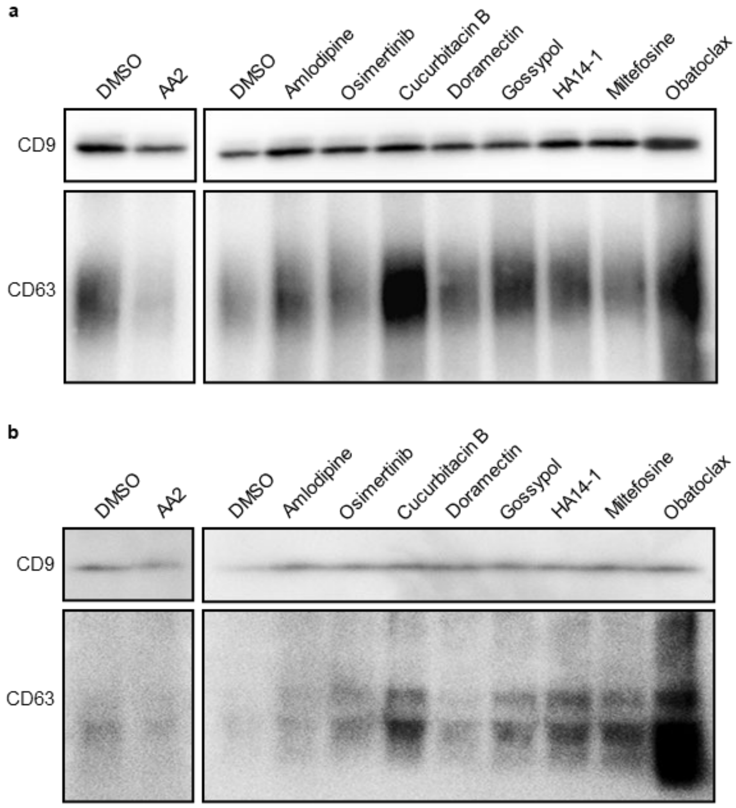

Supplementary Figure S2. Western blot analysis of EV secretion induced by the EV regulators. (**a**, **b**) U87MG cells were treated with 10 μM AA2, 7 μM amlodipine, 2 μM osimertinib, 1 μM cucurbitacin B, 2 μM doramectin, 10 μM gossypol, 15 μM HA14-1, 20 μM miltefosine, or 1 μM obatoclax for 24 h. The conditioned medium was separated by serial centrifugation to recover 10K sup. (**a**) EVs isolated from the 10K sup by the MagCapture Exosome Isolation Kit PS were subjected to western blot with anti-CD9 or anti-CD63 antibody. (**b**) The 10K sup was lysed in 2× sample buffer and subjected to western blot with anti-CD9 or anti-CD63 antibody. Full-length blots can be found in the supplementary information.

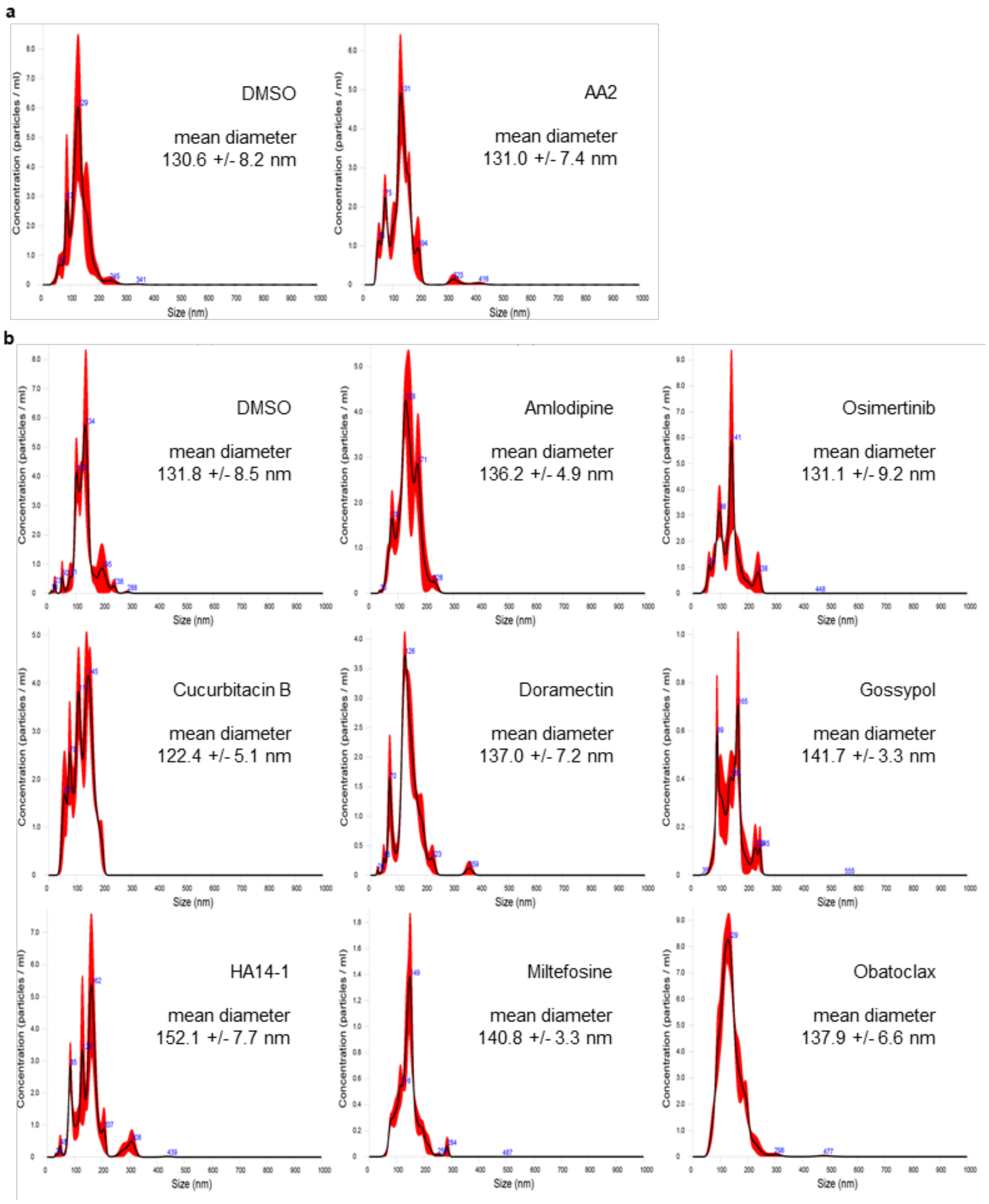

Supplementary Figure S3. Size distribution of EVs from U87 cells treated with EV regulators. (**a**, **b**) U87MG cells were treated with 10 μM AA2 (**a**), 7 μM amlodipine, 2 μM osimertinib, 1 μM cucurbitacin B, 2 μM doramectin, 10 μM gossypol, 15 μM HA14-1, 20 μM miltefosine, or 1 μM obatoclax (**b**) for 24 h. The conditioned medium was separated by serial centrifugation to recover 10K sup. The 10K sup was subjected to NTA.

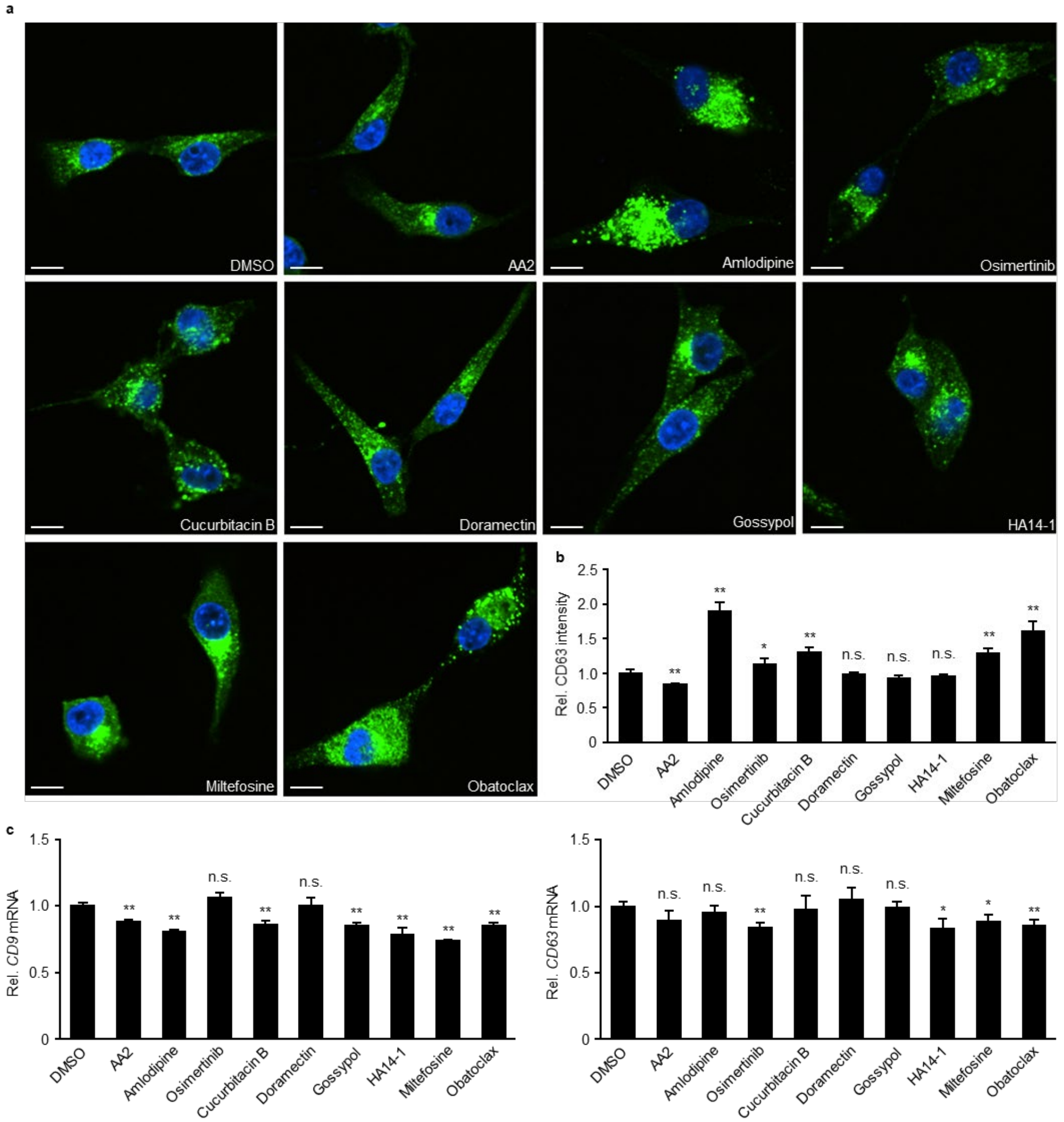

Supplementary Figure S4. Expression of CD63 in U87MG cells treated with EV regulators. (**a**, **b**) U87MG cells seeded on a glass chamber (Watson, Tokyo, Japan) were cultured for 24 h in media containing EV regulators. After fixing the cells with 4% paraformaldehyde and permeabilizing with ice-cold acetone, the cells were stained with Alexa Fluor 488-conjugated anti-CD63 antibody (BioLegend, Cat #353037, ×500) and mounted with VECTASHIELD HardSet Antifade Mounting Medium with DAPI (Vector laboratories, Burlingame, CA). Cells were imaged using an ECLIPSE Ti2 microscope (Nikon, Tokyo, Japan) and confocal imaging system Dragonfly 500 (Andor-Oxford Instruments, Abingdon-on-Thames, United Kingdom) at ×60 magnification. Scale bars represent 10 µm. (**a**). The intensity of CD63 was quantified using ImageJ (NIH, Bethesda, MD) in more than 40 cells (**b**). (**c**) Total RNA was extracted from U87MG cells after 24 h of treatment with EV regulators. Then, cDNA was reverse transcribed from total RNA, and the expression of *CD9*, *CD63*, and *β-actin* was detected using

RTqPCR. Primers, *CD9*-Fw; 5′-CCTGCTGTTCGGATTTAACTTCA-3′, *CD9*-Rv; 5′-TGGTCTGAGAGTCGAATCGGA-3′; *CD63*-Fw; 5′-CAGTGGTCATCATCGCAGTG-3′, *CD63*-Rv; 5′-ATCGAAGCAGTGTGGTTGTTT-3′; *β-actin*-Fw; 5′-CATGTACGTTGCTATCCAGGC-3′, *β-actin*-Rv; 5′-CTCCTTAATGTCACGCACGAT-3′. *$p < 0.05$, **$p < 0.01$; n.s., not significant vs. DMSO, Student's t-test.

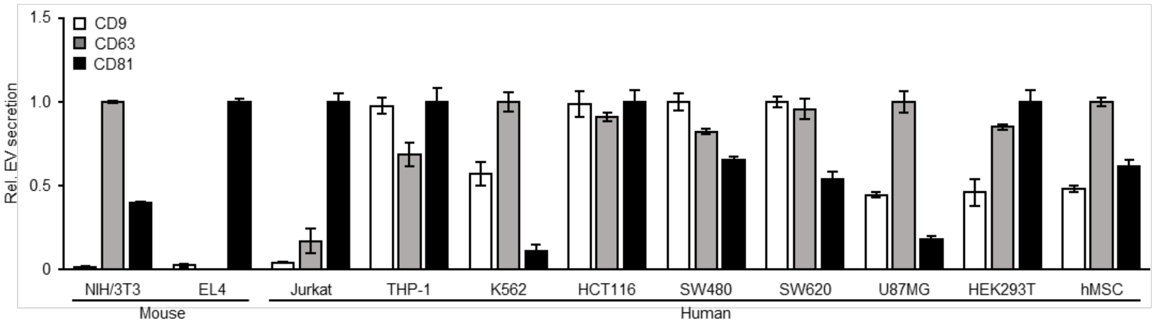

Supplementary Figure S5. Comparison of EV markers. Cells were cultured for 24 h followed by 10K sup collection. The 10K sup was subjected to TIM4-CD9, TIM4-CD63, and TIM4-CD81 ELISAs. Each sample's absorbance was normalized to the absorbance of the highest marker in each cell line.

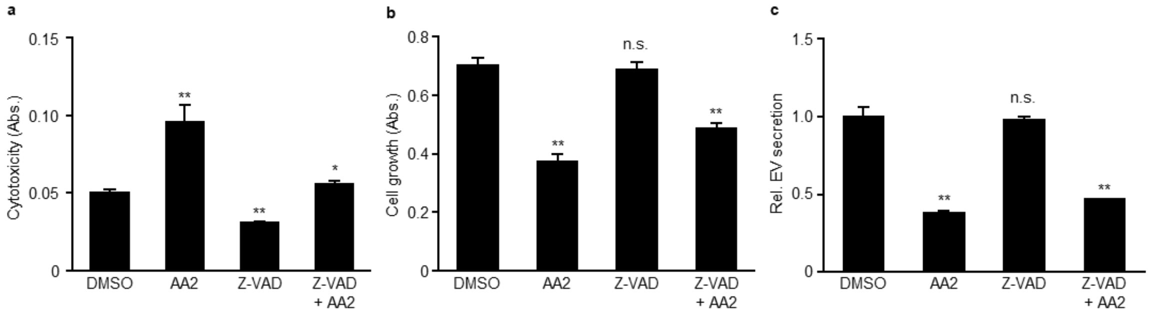

Supplementary Figure S6. AA2 inhibited EV secretion independent of caspase 3. (**a**–**c**) Jurkat cells were pre-treated with 0 or 50 µM Z-VAD(OMe)-FMK for 3 h, and then with 0 or 5 µM AA2 for 24 h. Cytotoxicity and cell growth were determined using lactate dehydrogenase (LDH) (**a**) and WST-8 (**b**) assays. (**c**) Secreted extracellular vesicles (EVs) were determined using a TIM4-CD81 ELISA. **p* < 0.05, ***p* < 0.01; n.s., not significant vs. DMSO, Student's *t*-test.

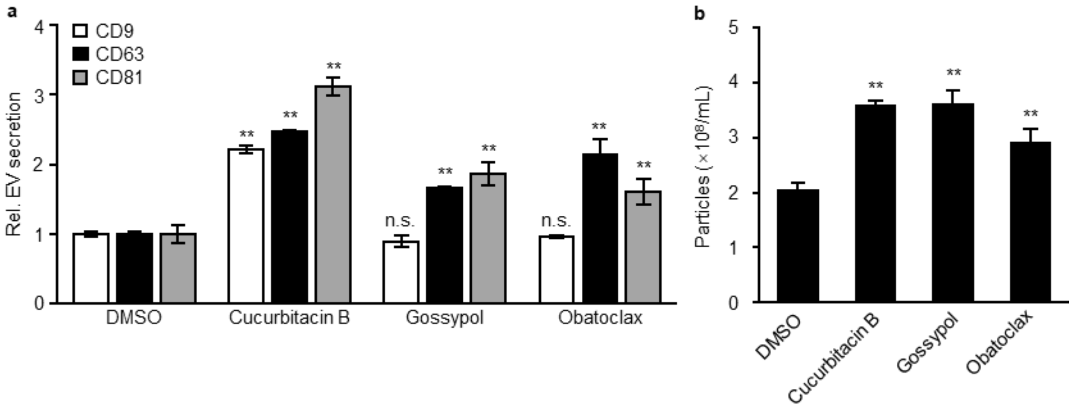

Supplementary Figure S7. Cucurbitacin B, gossypol, and obatoclax induced EV secretion in HEK293 cells. (**a**, **b**) HEK293 cells were treated with 1 µM cucurbitacin B, 0.3 µM gossypol, or 0.03 µM obatoclax for 24 h and then secreted EVs were determined using TIM4-CD9 and TIM4-CD63 ELISAs (**a**) or NTA (**b**). **p* < 0.05, ***p* < 0.01; n.s., not significant vs. DMSO, Student's *t*-test.

Full-length blots

Figure 1g

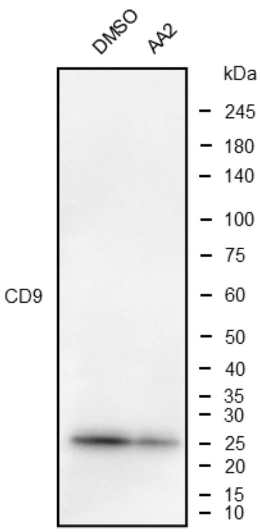

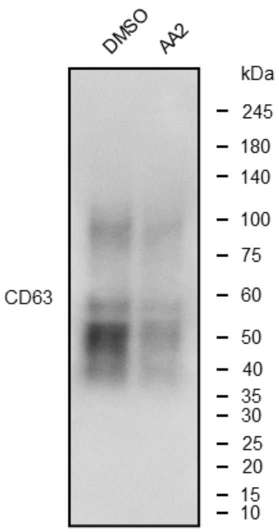

Figure 1l

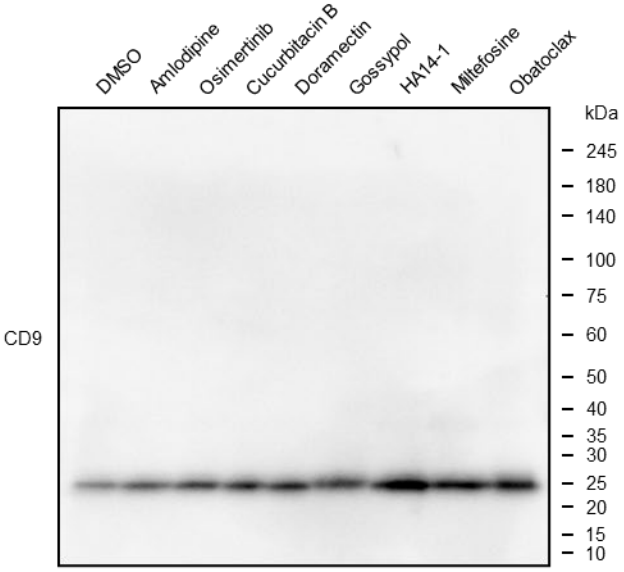

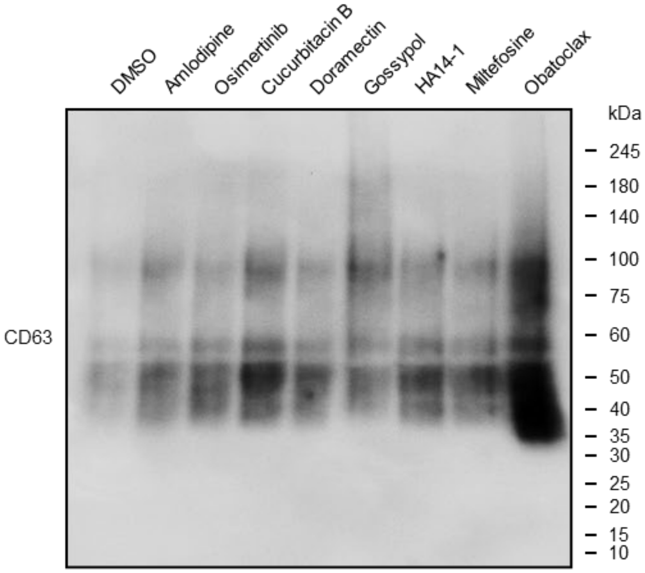

Figure 2j

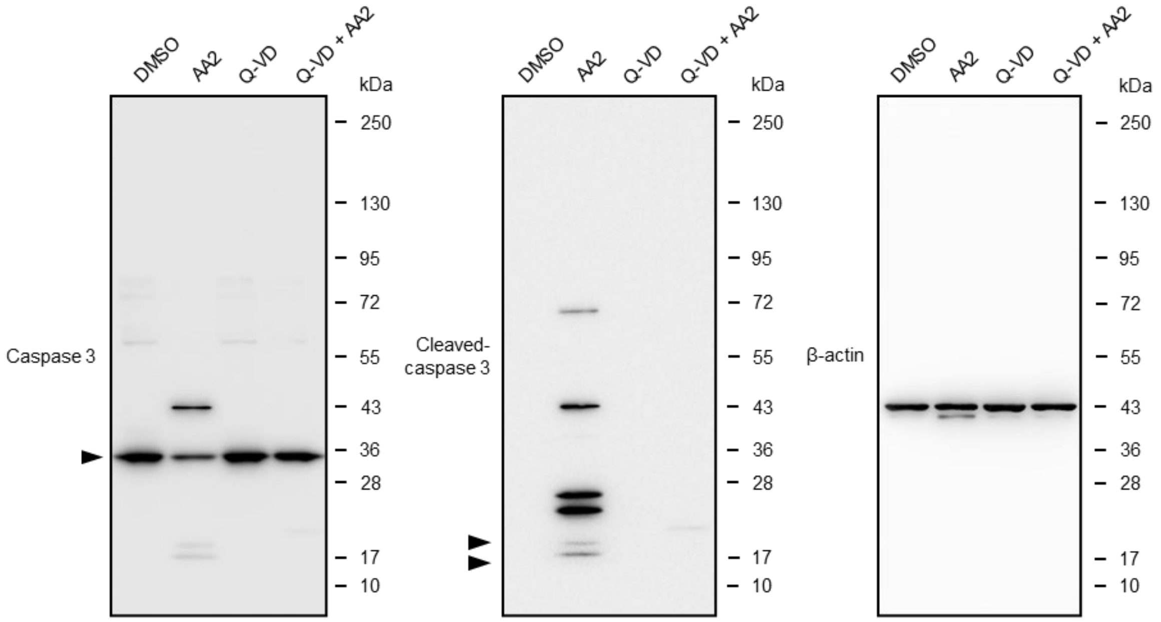

Figure 2l

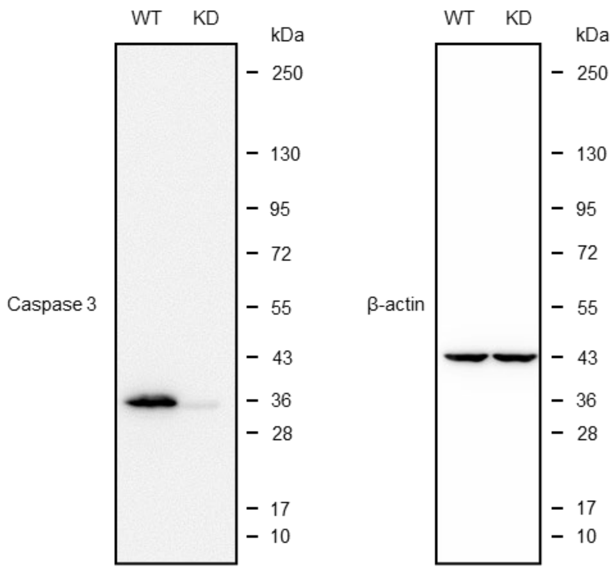

Figure 4b

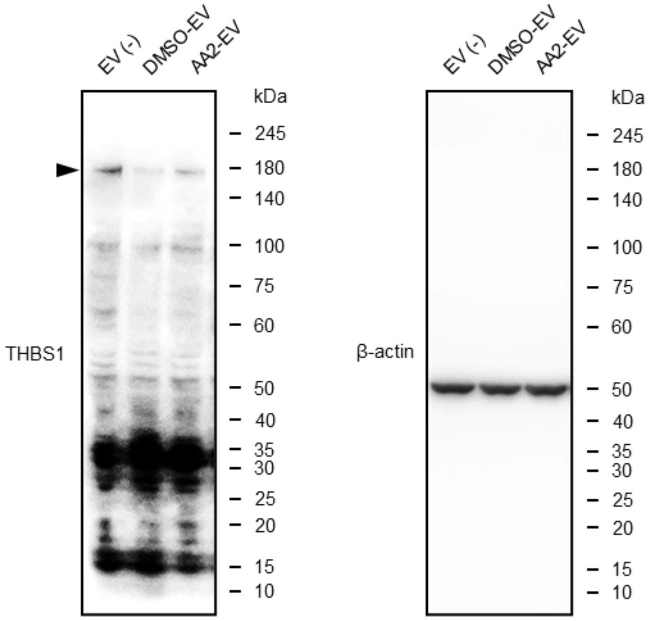

Figure 4d

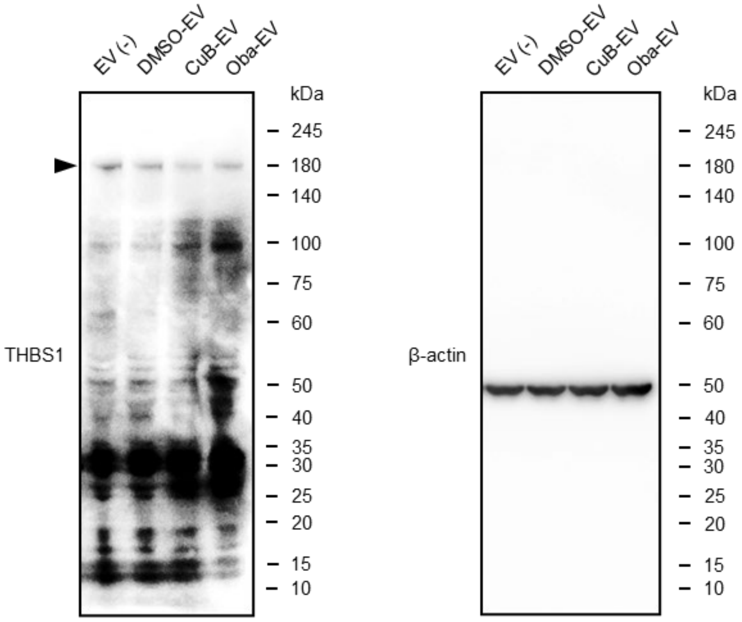

Supplementary Figure S2a

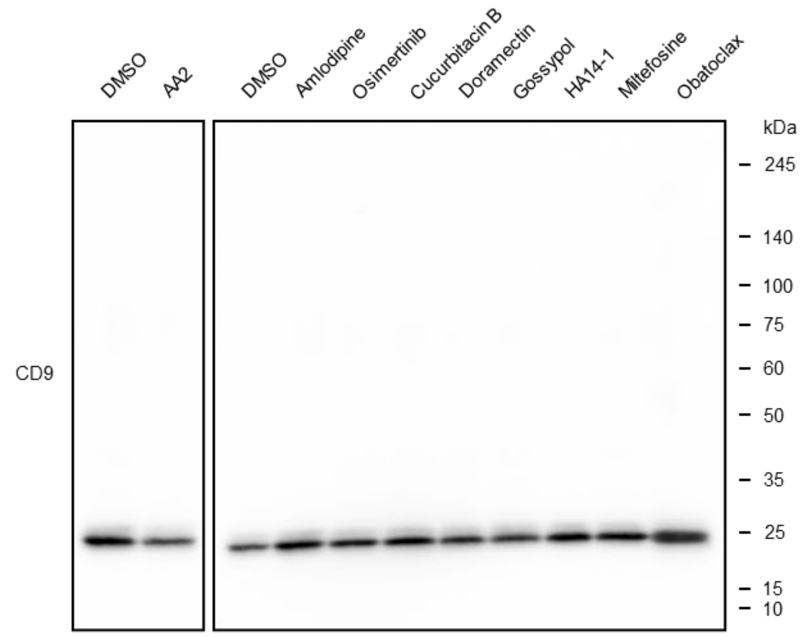

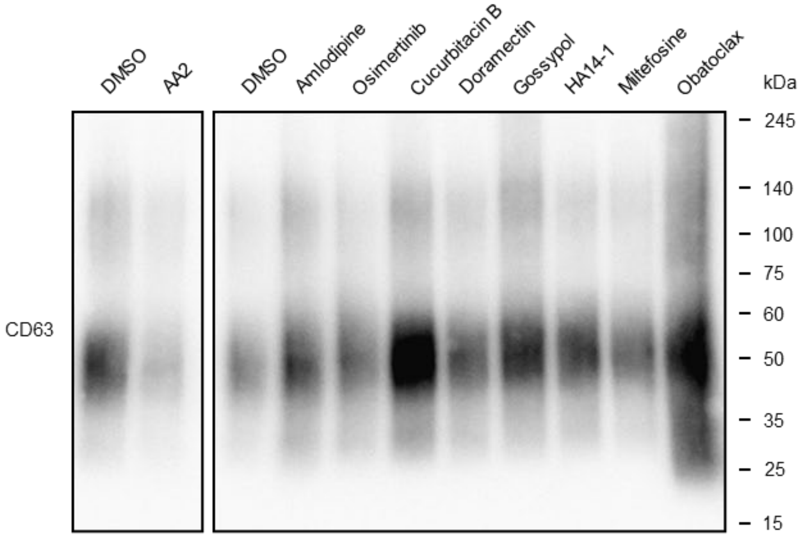

Supplementary Figure S2b

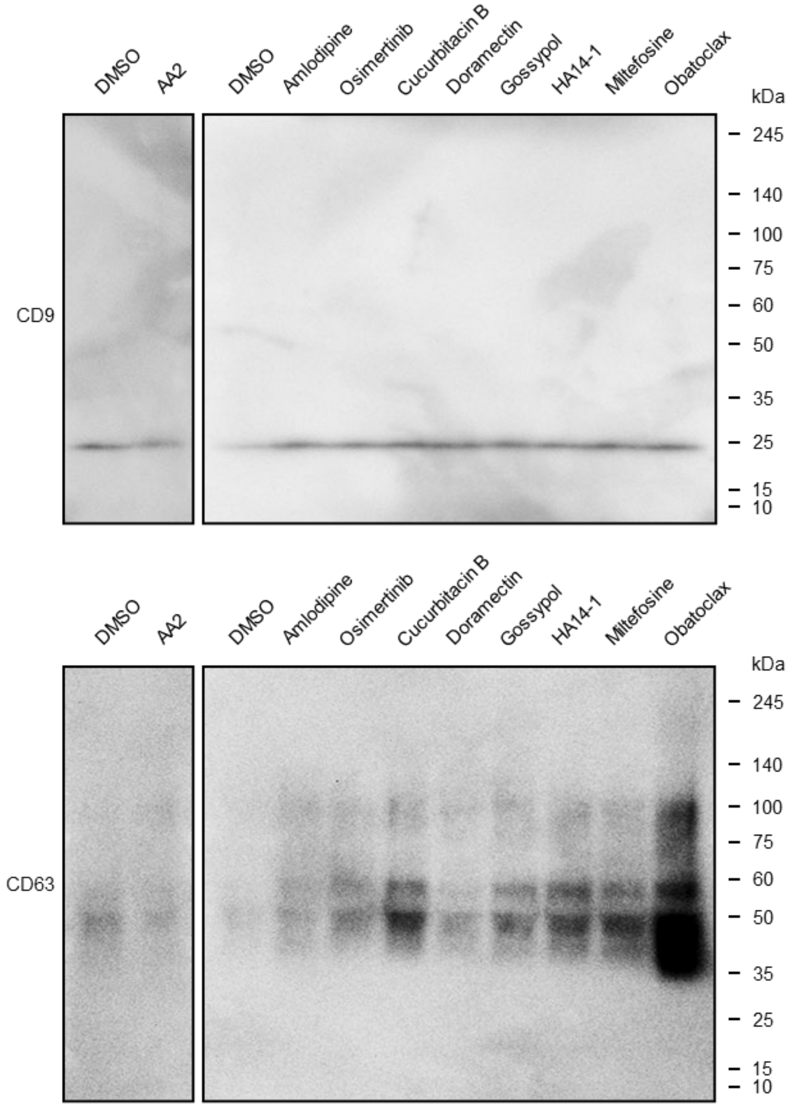

Supplement: Supplementary file 1 — Supplementary Information. [file 41598_2021_92860_MOESM1_ESM.pdf]
